# The impact of *Helicobacter pylori* infection and eradication therapy containing minocycline and metronidazole on intestinal microbiota

**DOI:** 10.1186/s12866-022-02732-6

**Published:** 2022-12-29

**Authors:** Meng-Yan Cui, Zhen-Yu Cui, Meng-Qi Zhao, Meng-Jie Zhang, Qiao-Li Jiang, Jing-Jing Wang, Lun-Gen Lu, Ying-Ying Lu

**Affiliations:** 1 Department of Gastroenterology, Shanghai General Hospital, Shanghai Jiao Tong University School of Medicine, Shanghai, 201620 China; 2 Department of Gastroenterology, Jiading Branch of Shanghai General Hospital, Shanghai Jiao Tong University School of Medicine, Shanghai, 201800 China; 3 Shanghai Key Laboratory of Pancreatic Diseases, Institute of Translational Medicine, Shanghai General Hospital, Shanghai Jiao Tong University School of Medicine, Shanghai, 201620 China

**Keywords:** Intestinal microbiota,, *Helicobacter pylori*, Eradication therapy, Minocycline, Metronidazole

## Abstract

**Background:**

*Helicobacter pylori* (*H. pylori*) infection is associated with remodeling of gut microbiota. Many studies have found *H. pylori* infection and eradication therapy can alter the gut microbiota. However, few studies explored the impact of eradication therapy containing minocycline and metronidazole on gut microbiota.

**Aim:**

The objective of the present study was to explore the changes of gut microbiota after *H. pylori* infection. Besides, learn more about the dynamic changes of gut microbiota during different stages of eradication treatment containing minocycline, metronidazole, bismuth agents and proton pump inhibitors.

**Methods:**

Sixty stool samples from the patients with *H. pylori* infection before eradication, 14 and 42 days after eradication, and ten stool samples from non-infected individuals were collected. Subsequently, we performed 16S rRNA gene amplicon sequencing to analyze these samples, and the results were evaluated by using alpha diversity, beta diversity and microbial composition analyses. Phylogenetic Investigation of Communities by Reconstruction of Unobserved States was also used to predict the metabolic pathways according to the Kyoto Encyclopedia of Genes and Genomes database.

**Results:**

The alpha and beta diversity of the microbiota changed significantly in *H. pylori* infected individuals, but returned to baseline 42 days after eradication therapy. At the genus level, the abundances of *Bacteroidetes*, *[Ruminococcus]_gnavus_group*, *Ruminococcaceae_Incertae_Sedis*, *Tuzzrealla*, *Butyricicoccus* were significantly lower in the *H. pylori* infected group. Bacterial abundance was also dynamically changing during eradication treatment. In addition, PICRUST analysis found the levels of uronic acid metabolism, uncharacterized transport system, and biosynthesis of unsaturated fatty acids were higher in *H. pylori* infected individuals than in the non-infected group.

**Conclusions:**

Intestinal microbiota diversity, composition, functional predictions altered significantly after *H. pylori* infection, and gradually returned to healthy control levels after the application of eradication therapy containing minocycline and metronidazole in one month and a half.

**Supplementary Information:**

The online version contains supplementary material available at 10.1186/s12866-022-02732-6.

## Importance

*Helicobacter pylori* infection is associated with remodeling of intestinal microbiota. Many studies have found *H. pylori* infection and eradication therapy can alter the gut microbiota. However, few studies explored the impact of eradication therapy containing minocycline and metronidazole on gut microbiota. Sixty stool samples from the patients with *H. pylori* infection before eradication, 14 and 42 days after eradication, and ten stool samples from non-infected individuals were analyzed. We found that the intestinal microbiota was changed after *H. pylori* infection and during different stages of eradication treatment with minocycline and metronidazole. The quadruple eradication therapy containing minocycline, metronidazole had not disturbed the intestinal microbiota, and made the intestinal microbiological ecology back to the healthy state instead. This is the first study to focus on the effects of the minocycline regimen on the gut microbiota. Besides, compared with previous studies, minocycline regimen has less impact on gut microbiota than other treatments. This regime may be prospecting in the future clinical applications.

## Introduction

*Helicobacter pylori* (*H. pylori*) infection is one of the most common bacterial infections, affecting approximately 50% of the global population [1]. Based on regional prevalence estimates, there were still approximately 4.4 billion people infected with *H. pylori* worldwide [2]. *H. pylori* not only can lead to gastric diseases, such as peptic ulcer, atrophic gastritis and gastric cancer, but also associated with disease extragastric, including nonalcoholic fatty liver disease, primary sclerosing cholangitis [3–7]. Therefore, eradication of *H. pylori*, especially in high-risk groups of gastric cancer, has become a global consensus [8].

According to the *"Sixth National Guidelines for the Treatment of Helicobacter pylori Infection 2022"*, the bismuth quadruple regimen is the initial and re-treatment regimen for *H. pylori* infection. In addition to proton pump inhibitors (PPI) and bismuth, the following antimicrobial drug combinations are suggested, such as amoxicillin combined with clarithromycin, amoxicillin combined with levofloxacin, tetracycline combined with metronidazole, amoxicillin combined with metronidazole, and amoxicillin combined with tetracycline (Strong recommendation, moderate-quality evidence). Tetracycline combined with metronidazole is the only non-penicillin regimen, which is especially suitable for the patients allergic to penicillin. Since tetracycline has many side effects and has been gradually withdrawn from clinical application, second-generation tetracyclines such as minocycline have been widely used in clinical practice instead of tetracycline. Besides, in the guideline, experts propose that semi-synthetic tetracycline can be used in eradication treatment instead of tetracycline. Therefore, the quadruple anti-*H. pylori* regimen consisting of minocycline and metronidazole is currently recognized as a first-line treatment regimen.

Previous research reported that the rate of successful eradication via two-week administration of minocycline regimen in *H. pylori* infected individuals was ≥ 85% (95% CI, 77.1–95.1%) without severe side-effects [9]. Based on the current epidemiological investigation, the reported resistance for minocycline was below 10% all over the world [10]. It can be seen that this is a regime with the advantages of high eradication rate and low drug resistance rate. However, it has not received much attention from clinicians, so we focused on this regimen to explore the impact on human body.

In humans, the intestinal microbiota is a complex and dynamic ecosystem that has coevolved with its host [11]. During birth, bacteria colonize the infant’s gut rapidly, which was from the mother and the surrounding environment [12]. In adults, the intestinal microbiota remains dynamic balance slightly fluctuating around baseline [13]. The gut microbiota has been corroborated as a core regulator of host metabolism, which is involved in regulating various metabolic processes, including energy homeostasis, glucose metabolism, and lipid metabolism [14]. Besides, the gut microbiota is critical for maintaining normal gastrointestinal tract, immune functions, and efficient digestion of nutrients [15–17].

Changes and dysbiosis in intestinal microbiota composition may induce the pathogenesis of various disorders. *H. pylori* is the major risk factor for gastric cancer development, which may be related to the dysbiosis of the gastrointestinal tract caused by *H. pylori*. Research has shown that *H. pylori* colonization and intestinal microbiota work together to promote the occurrence of *H. pylori*-related gastric cancer [18]. On the other hand, antibiotics used to eradicate *H. pylori* might also affect intestinal microbiota proliferation and alter microbial diversity. Short-term treatment in humans with a single dose of oral antibiotics alters the gut microbiota for as long as 4 weeks before it tends to recover its original composition [19]. In a multi-center study, it was found that the intestinal microbial species decreased by more than 30% in the patients treated with eradication therapy within 1 year [20]. A previous study has shown that after applying the eradication therapy containing vonoprazan, amoxicillin, and clarithromycin, it takes 3 months for the diversity and structure of the intestinal microbiota to recover to the baseline of non-infected individuals [21]. Nevertheless, since different antibiotics may have different effects on intestinal microbiota, uncertainties remain in the outcomes following the application of the quadruple regimen consisting of minocycline combined with metronidazole, bismuth agents, and proton pump inhibitors.

In this study, we explored the changes of intestinal microbiota after *H. pylori* infection and during different stages of eradication treatment with minocycline, metronidazole, bismuth and PPI.

## Material and methods

### Study design

This study designed to explore the changes of intestinal microbiota between non-infected individuals and *H. pylori* infected individuals. Besides, learn more about the changes of intestinal microbiota during different stages of eradication treatment containing minocycline, metronidazole, bismuth agents, and proton pump inhibitors. This prospective study, which was approved by the Institutional Review Board of Shanghai General Hospital, collected samples from November 2020 to May 2021. Written informed consent was obtained from all individuals before enrollment. All the authors had access to the study data and had reviewed and approved the final manuscript.

### Study population

*H. pylori* infected individuals were recruited in outpatient department of the Jiading branch of Shanghai General Hospital. *H. pylori* infected individuals who entered the study met the following criteria: men or women aged 25–50, diagnosed with *H. pylori* infection, and had never received anti-*H. pylori* treatment. *H. pylori* infection was confirmed by at least one positive results of histological examination, or rapid urease test during gastroscopy, or [13]C urea breath test.

Patients with any one of the following conditions were excluded: those who had used antibiotics, bismuth, or PPI during the past 2 weeks; those who used eradication therapy before; those who had used non-steroidal anti-inflammatory drugs (NSAIDs) during the last 4 weeks; those who had a history of allergic reactions to metronidazole or tetracycline; those who had clinically significant hepatic, renal, cardiovascular, respiratory, endocrine, or central nervous system disorders; those who were pregnant or nursing mothers; and those who had a history of gastrointestinal surgery.

Non-infected controls were men or women aged 25–50, recruited from healthy residents in Shanghai area. Non-infected controls were confirmed through [13]C urea breath test, and had never received eradication treatment before. Besides, they had never been diagnosed with severe digestive disorders, such as acute or chronic pancreatitis, cholecystitis or cholangitis, and hepatic insufficiency.

### Procedures

A complete medical history and demographic were obtained from each individual, including age, gender, BMI, history of smoking, alcohol on enrollment. All of the enrolled individuals, including *H. pylori* infected individuals and non-infected controls, were asked to keep a stool sample as a baseline before trial beginning. Then *H. pylori* infected individuals received a 14-day quadruple eradication therapy (rabeprazole 10 mg twice daily, metronidazole 400 mg three times a day, minocycline 100 mg twice a day, and bismuth potassium citrate capsules 220 mg twice a day for 14 days), and were asked to return at the end of eradication therapy and 4 weeks after the end of eradication therapy to take a [13]C urea breath test to assess post-treatment *H. pylori* status and keep stool samples.

### 16S rRNA gene amplification and sequencing by MiSeq

Using the E.Z.N.A.® Stool DNA Kit (Omega Bio-tek, Inc., GA) to extracted bacterial DNA. All the steps followed the manufacturer's instructions.

Using the bacterial universal primer F1 and R2 to amplify the V3-V4 region of the bacterial 16S ribosomal RNA gene (5’- CCTACGGGNGGCWGCAG -3’ and 5’-GACTACHVGGGTATCTAATCC-3’). The PCR reaction was performed in the T100™ Thermal Cycler PCR system (Bio-Rad Laboratories, Inc., USA). Programs were as follows: denaturing at 95 °C for 3 min, then 21 cycles at 94 °C for 0.5 min (denaturation), annealing at 58 °C for 0.5 min, 0.5 min at 72 °C (elongation), and finally extending for 5 min at 72 °C.

Products from different samples were indexed and mixed by using the Misq platform (Illumina Inc., USA) according to the manufacturer's instructions for sequencing.

### Bioinformatics and statistical analysis

Use USEARCH (version 11.0.667) to extract clean data from raw data. The mass filter sequences were clustered into unique sequences, and the sequences with mass filtering were sorted by UPARSE according to the UPARSE OTU analysis pipeline, and the singleton sequence was omitted. After removing the chimeric sequence by using UPARSE (version 7.1), the operational classifier (OTU) was classified based on 97% similarity and annotated using the SILVA Reference Database (SSU138). Evaluate alpha diversity metrics using Mothur v1.42.1 (ACE Estimator, Chao 1 Estimator). Bray–Curtis dissimilarity was calculated in QIIME.Principal coordinate analysis (PCoA) plots and PERMANOVA were generated in R (version 3.6.0) package vegan 2.5–7. The linear discriminant analysis (LDA) effect size (LEfSe) was used to detect differences in abundance between groups (lefse 1.1). PICRUSt2 (v2.4.1) was used to predict functional abundances based on 16S rRNA gene sequences.

## Results

### Baseline demographics and study follow-up

**Fig. 1:**
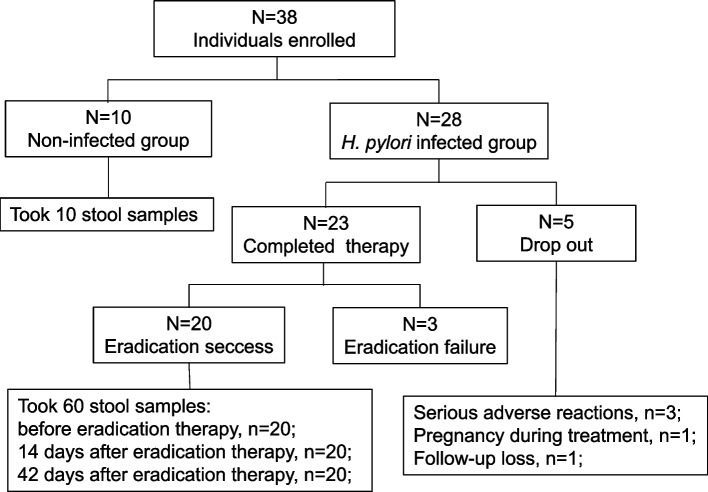
The design of the individuals enrolled

A total of 38 patients were initially screened for the study, including 28 *H. pylori* infected individuals and 10 non-infected controls. Twenty-three *H. pylori* infected individuals completed eradication therapy, while 5 patients in *H. pylori* infected group withdrew from the study for various reasons. Among the patients who completed the 14-days eradication therapy, 20 patients had negative result of [13]C breath test, while the other 3 patients were positive. We sequenced stool samples from patients with successful eradication and non-infected individuals only. A total of 70 stool samples were collected from 20 *H. pylori* infected individuals and 10 non-infected controls. (Fig. 1) Baseline characteristics were similar among both groups. (Table 1).

**Table 1:** Baseline characteristics of the individuals enrolled

Variable	*H. pylori* infected group	non-infected group	*P* value
	**(*N* = 20)**	**(*N* = 10)**	
**Age (mean ± SD)**	37.60 ± 5.83	39.10 ± 3.90	0.470
**Gender**			0.255
** Female**	13(65%)	4(40%)	
** Male**	7(35%)	6(60%)	
**BMI**	21.84 ± 1.52	21.66 ± 1.75	0.768
** History of smoking; yes**	5(25%)	2(20%)	0.760
** History of alcohol; yes**	3(15%)	1(10%)	0.704

### Alpha diversity analysis of non-infected individuals and H. pylori infected individuals at different treatment stage

**Fig. 2:**
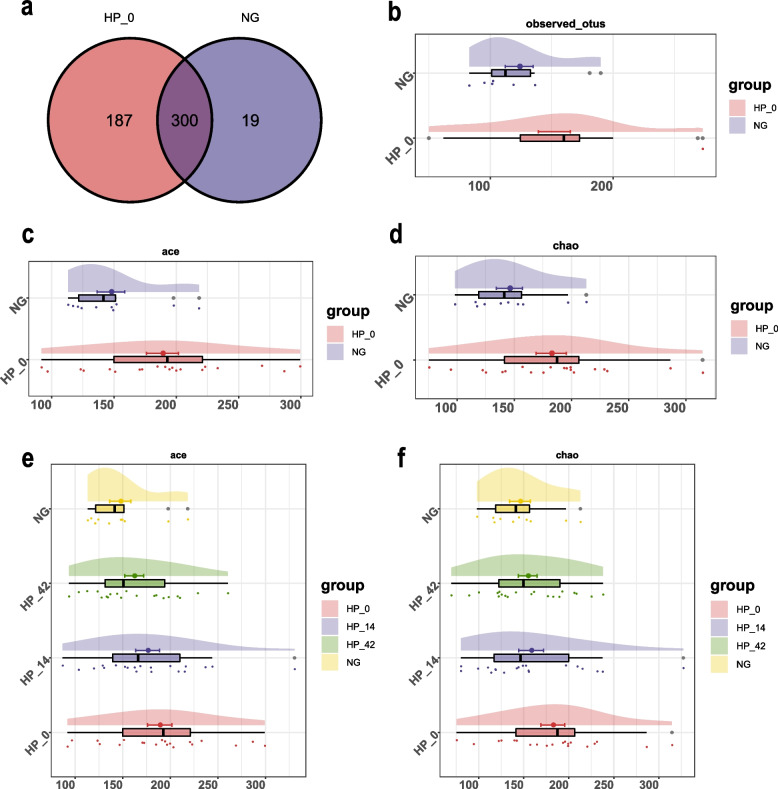
Operational Taxonomic Units clustering and alpha diversity analysis of intestinal microflora. **a** Venn diagram demonstrates the shared and unique Operational Taxonomic Units (OTUs) in *H. pylori*-infected group and non-infected controls. **b** The OTUs in the single sample in *H. pylori*-infected group and non-infected controls. **c** Ace index of *H. pylori*-infected group and non-infected controls. **d** Chao index of *H. pylori*-infected group and non-infected controls. **e** Ace index of non-infected controls and *H. pylori*-infected group at different treatment stage. **f** Chao index of non-infected controls and *H. pylori*-infected group at different treatment stage

After dividing by 97% similarity, a total of 506 operational taxonomic units (OUTs) were obtained in non-infected group and *H. pylori* infected group, the Venn diagram showed that 300 of the 506 OTUs were shared by both groups, whereas 187 OTUs were unique to the *H. pylori* infected group, and 19 were specific to the non-infected group (Fig. 2a). The resulting rarefaction curves indicate that the microbial richness of the sampled guts was near saturation at the applied sequencing depth, which was sufficient to identify most of the bacterial community members in each individual. The Shannon curve based on the OTUs was already flat, indicating that our sequencing depth was already adequate (Supplementary Fig. 1). Alpha diversity reflects the diversity of an ecological communities within a sample. We choose ‘ace index’ and ‘chao index’, which including information about the number of different OTUs, to access the within-habitat diversity.

As shown in Fig. 2b-d, the alpha diversity of the intestinal microflora was higher for the *H. pylori* infected group than it was for the non-infected group. The alpha diversity estimations revealed significantly higher scores in *H. pylori* cases compared to controls for ‘ace index’ (188.85 ± 12.84 vs 147.52 ± 11.10, *P* = 0.049). Although the ‘chao’ scores of *H. pylori* cases were also higher compared to controls, there was just a trend of difference (182.32 ± 13.18 vs 145.79 ± 11.45, *P* = 0.074).

As eradication treatment time went by, the ‘ace index’ and ‘chao index’ of the *H. pylori* infected individuals showed a downward trend, and the data gradually approached the non-infected controls. Finally, it was confirmed that there was no statistical difference between 42 days after eradication therapy group and non-infected group no matter in the ‘ace index’ (161.98 ± 9.98 vs 147.52 ± 11.10, *P* = 0.448) or in the ‘chao index’ (154.50 ± 10.42 vs 145.79 ± 11.45, *P* = 0.588), which was reflected in Fig. 2e-f.

### Beta diversity analysis of non-infected individuals and H. pylori infected individuals at different treatment stage

**Fig. 3:**
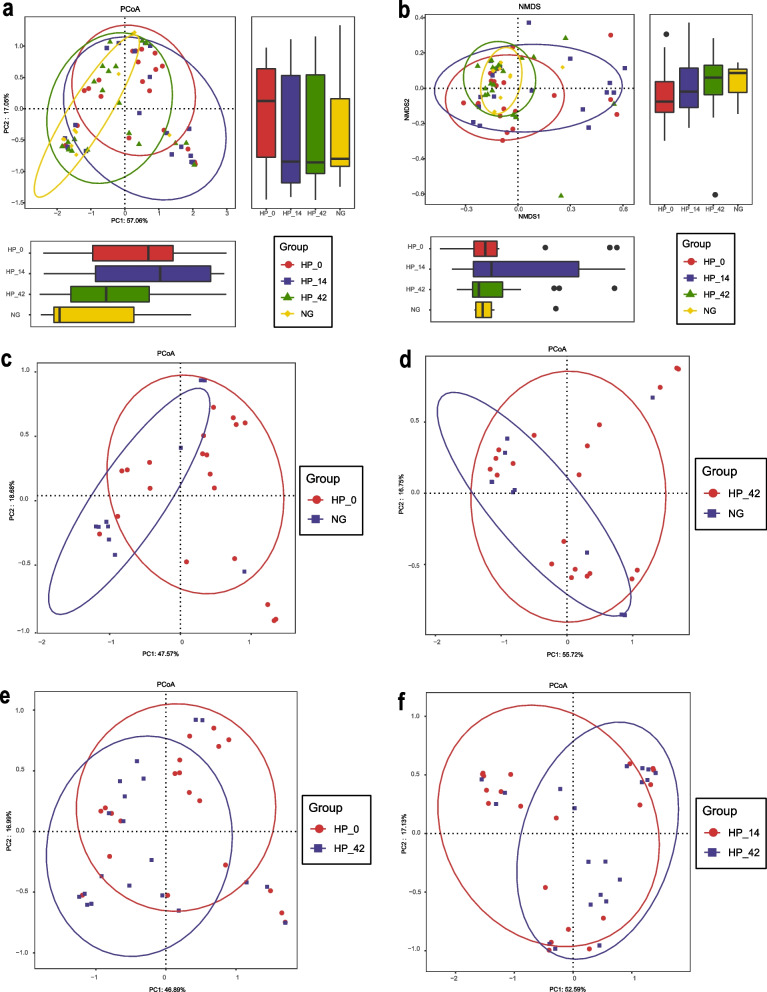
Beta diversity analysis of intestinal microflora based on Operational Taxonomic Units levels. **a** Principal coordinates analysis (PCoA) based on Bray–Curtis dissimilarity of intestinal microflora in non-infected controls and *H. pylori* infected individuals at different treatment stage. The box plots on the right and the bottom show the differences in the distribution of microbial communities based on the first and second principal coordinates of the samples in different groups. **b** Nonmetric multidimensional scaling analysis (NMDS) based on Bray–Curtis distances of intestinal microflora in non-infected controls and *H. pylori* infected individuals at different treatment stage. The box plots on the right and the bottom show the differences in the distribution of microbial communities based on the first and second principal coordinates of the samples in different groups. **c** PCoA based on Bray–Curtis distances of intestinal microflora in non-infected controls and *H. pylori* infected individuals before treatment. **d** PCoA based on Bray–Curtis distances of intestinal microflora in non-infected controls and *H. pylori* infected individuals 42 days after eradication therapy. **e** PCoA based on Bray–Curtis distances of intestinal microflora in *H. pylori* infected individuals before treatment and 42 days after eradication therapy. **f** PCoA based on Bray–Curtis distances of intestinal microflora in *H. pylori* infected individuals 14 days and 42 days after eradication therapy

Beta diversity was used to access how samples differ from each other. We used the commonly applied Bray–Curtis analysis which was calculated based on the evolutionary information between individual sample sequences to compare whether samples had significant microbial community differences. Figure 3 showed the result of a principal coordinate analysis (PCoA) and a non-metric multidimensional scaling analysis (NMDS) based on Bray–Curtis dissimilarity.

There was a trend of difference in the bacterial composition of *H. pylori* infected patients and non-infected controls at baseline according to PCoA (R^2^ = 0.054, *P* = 0.087) (Fig. 3c).

As we can see in Fig. 3a-b, with the progress of eradication treatment, the beta diversity of intestinal flora in patients with *H. pylori* infection gradually return to healthy control levels. The composition of the microbiota in the 42 days after eradication therapy group and non-infected group was similar, and the Adonis analysis also showed that there was no significant difference in the overall composition of the flora between the two groups (R^2^ = 0.031, *P* = 0.518) (Fig. 3d). Besides, there was some extent of difference in microflora within *H. pylori* infected individuals before treatment had some extent of difference with 42 days after eradication therapy group (R^2^ = 0.038, *P* = 0.106) (Fig. 3e).

There was also a trend of difference in the bacterial composition of 14 days after eradication therapy group and 42 days after eradication therapy group (R^2^ = 0.044, *P* = 0.064) (Fig. 3f). And the flora in 42 days after eradication therapy group was closer to the healthy control level, which might be related to the self-regulation of the gut microbiota.

### Microflora composition in non-infected group and H. pylori infected group at different treatment stage

**Fig. 4:**
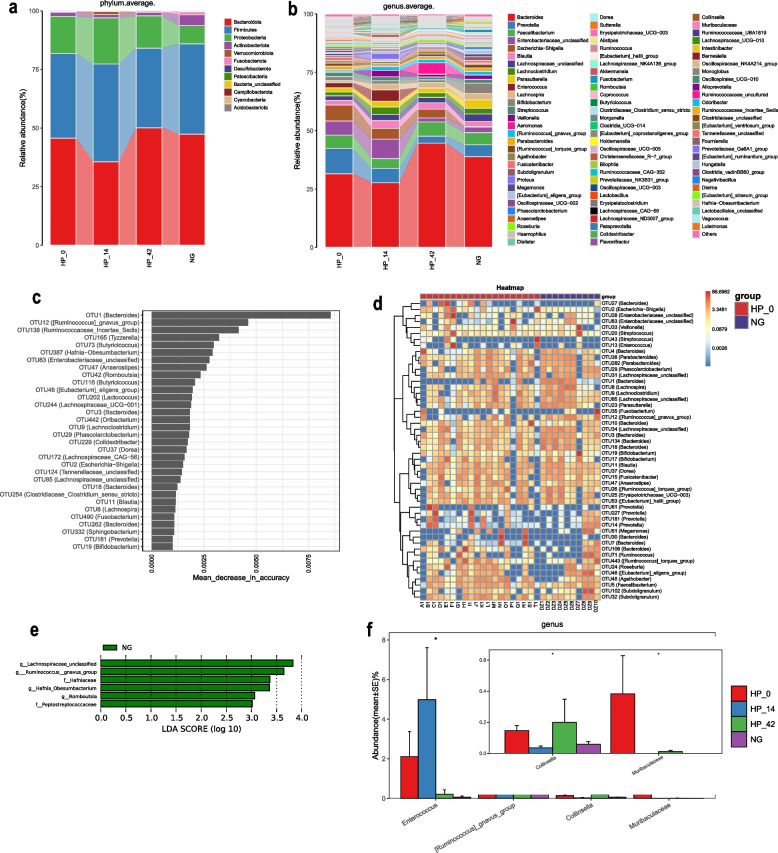
The composition of intestinal microflora, and the comparison in each group. **a** Flora composition at the phylum level. **b** Flora composition at the genus level. **c** Bar depict showed random-forest analysis for screening differential microbiota between non-infected group and *H. pylori* infected group. **d** A heatmap analysis of the microbiomes between non-infected group and *H. pylori* infected group. **e** Bacteria selected by Lefse with significant differences between non-infected group and *H. pylori* infected individuals before treatment. **f** Kruskal–Wallis rank sum test to screen differences in the flora at the genus level in all the groups

A total of 13 phyla were detected by classifying the species of all OTUs. The average relative abundance of the microbiome at the phylum level is shown in Fig. 4a. At the phylum level, Bacteroidetes, Firmicutes and Proteobacteria were the dominant phyla in all of the group. According to the result of Kruskal–Wallis rank sum test, no significant difference among the groups was found at the phylum level.

The average relative abundance of the microbiome at the genus level is shown in Fig. 4b. At the genus level, the gut microbiota was dominated by *Bacteroides* in the non-infected group, followed by *Faecalibacterium*, *Prevotella*, and *Bifidobacterium* with proportions of 38.9%, 5.2%, 5.0%, 4.5%, respectively. Correspondingly, *Bacteroides* was the most dominant bacteria in *H. pylori* infected individuals before eradication treatment, followed by *Prevotella*, *Escherichia-Shigella*, and *Enterobacteriaceae_unclassified* with proportions of 31.6%, 10.8%, 6.1%, 6.8%, respectively. Besides, *Bacteroides* was also the most dominant bacteria in *H. pylori* infected individuals 14 days after treatment and 42 days after treatment. However, *Enterobacteriaceae_unclassified*, *Prevotella*, and *Enterococcus* were dominant in 14 days after treatment group. *Faecalibacterium*, *Aeromonas*, and *Escherichia-Shigella* were dominant in 42 days after treatment group.

### The comparison of microflora composition in non-infected group and H. pylori infected group at different treatment stage

The abundance of many bacteria decreased in the *H. pylori* infected individuals. As analyzed by the random forest, at the genus level, *Bacteroidetes*, *[Ruminococcus]_gnavus_group*, *Ruminococcaceae_Incertae_Sedis*, *Tuzzrealla*, *Butyricicoccus* were significantly lower in the *H. pylori* infected group than in the non-infected group (Fig. 4c-d). Similar results were also confirmed in the LEfSe analysis. As shown in Fig. 4e, *Lachnospiraceae_unclassified*, *[Ruminococcus]_gnavus_group*, *Hafniaceae*, *Hafnia_Obesumbacterium*, *Romboutsia*, *Peptostreptococcaceae* were decreased in the *H. pylori* infected group.

According to the Kruskal–Wallis rank sum test, it was found that the abundances of *Entercoccus* was significantly increased in *H. pylori* infected individuals after 14 days treatment, and decreased after 42 days treatment. And there was no significant difference between 42 days after treatment group and non-infected individuals. Although there was a significant difference in the abundance of *[Ruminococcus]_gnavus_group* in the random forest analysis, it did not fluctuate significantly in Fig. 4f. Besides, it was shown that *Muribaculaceae* only survived in *H. pylori* infected individuals before treatment and 42 days after treatment, and the abundance was significantly higher in *H. pylori* infected individuals before treatment (Fig. 4f).

We used MaAsLin (Multivariate Analysis by Linear Models) to perform correlation analysis on the microbiota data and age and gender. The result showed that intestinal microbiota was not related to the age or gender.

### Functional alterations of intestinal microbiomes in H. pylori infected individuals at different treatment stage

16S sequencing data was used for functional prediction based on the KEGG module database and KEGG pathway database, and a LEfSe was subsequently used to sort the different metabolic modules and pathways between the groups.

**Fig. 5:**
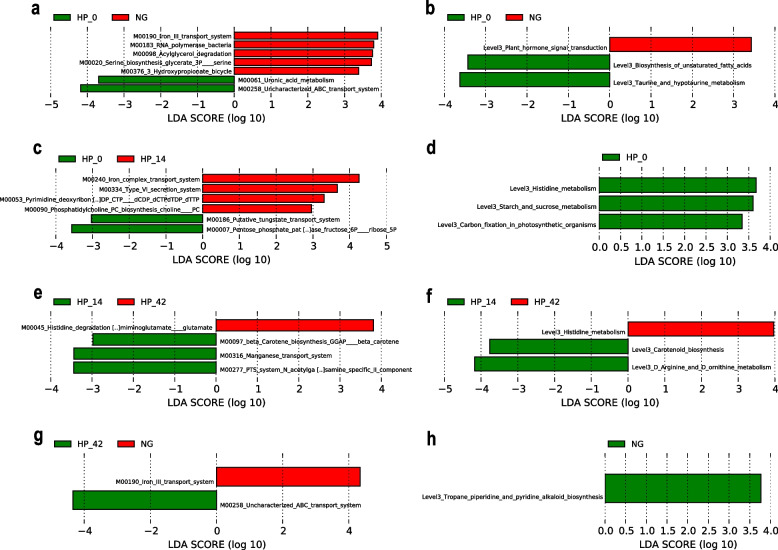
Predictions of the functional alteration to the intestinal microbiomes in each group. **a** Altered modules in patients with *H. pylori* infection. **b** Altered metabolic pathways in *H. pylori* infected individuals as shown by the histogram of the LDA scores. **c** Altered modules between *H. pylori* infected individuals before and after 14 days eradication treatment. **d** Altered metabolic pathways between *H. pylori* infected individuals before and after 14 days eradication treatment. **e** Altered modules between *H. pylori* infected individuals after 14 days and 42 days eradication treatment. **f** Altered metabolic pathways between *H. pylori* infected individuals after 14 days and 42 days eradication treatment. **g** Altered modules between non-infected individuals and *H. pylori* infected individuals after 42 days eradication treatment. **h** Altered metabolic pathways between non-infected individuals and *H. pylori* infected individuals after 42 days eradication treatment

The results demonstrated that iron transport system, RNA polymerase bacteria, acylglycerol degradation, Serine biosynthesis glycerate 3P serine, hydroxypropionate bicycle, plant hormone signal transduction were significantly higher in non-infected individuals compared to the *H. pylori* infected individuals. However, uronic acid metabolism, uncharacterized transport system, biosynthesis of unsaturated fatty acids, taurine and hypotaurine metabolism were all significantly higher in *H. pylori* infected individuals than in the non-infected group (Fig. 5a-b).

During the period of treatment, the functional predictions were always in dynamic changing. For instance, iron complex transport system, secretion system increased in *H. pylori* infected individuals after 14 days treatment. Besides, putative tungstate system, histidine metabolism, starch and sucrose metabolism, carbon fixation in photosynthetic organisms decreased after 14 days treatment (Fig. 5c-d). Histidine metabolism further increase over treatment time (Fig. 5e-f). Finally, after 42 days of treatment, the functional predictions no matter based on KEGG module or KEGG pathway showed fewer difference with non-infected group. Only iron transport system still lower than the non-infected group. Uncharacterized transport system, tropane piperidine and pyridine alkaloid biosynthesis were higher than non-infected group (Fig. 5g-h).

## Discussion

In our research, we explored the differences of intestinal microbiota between *H. pylori* infected individual and non-infected individuals, and illustrated the dynamic changes of intestinal microbiota during the different stages of eradication treatment with minocycline, metronidazole, bismuth agents and proton pump inhibitors. Few studies concerned on the minocycline regimen although it has been first-line treatment for *H. pylori* eradication in clinical. This is the first study focusing on the effect of minocycline regimen on intestinal flora.

The data of our research clearly demonstrated that the α-diversity significantly increased in *H. pylori* infected individuals. *H. pylori* infection is an infectious disease, and maybe that is why the α-diversity increased. Besides, there was a gradually decrease after eradication and was almost indistinguishable from the non-infected group at 6 weeks post-treatment, which was consistent with a previous systematic review of 24 articles [22]. There was also a trend of difference in β-diversity between *H. pylori* infected individuals and non-infected individuals, but little difference between *H. pylori* infected individuals after 42 days of eradication treatment and non-infected individuals. Both the α-diversity and β-diversity showed that eradication of *H. pylori* can promote the recovery of gastrointestinal microbiota in some extent.

There was a certain trend of difference of β-diversity between the *H. pylori* infected individuals after 14 days of eradication treatment and after 42 days of eradication treatment. In this period, the patients had completed eradication treatment and stopped taking drugs. Hence, changes in gut microbiota were not affected by eradication drugs, but were entirely self-regulated by microbiota. It was suggested that the gut microbiota can gradually return to normal level through self-regulation after key pathogenic factors removed, such as *H. pylori*.

Changes in the composition of the microbiota also influence our body. In our research, the dominant microbiota in each group did not differ significantly at the phylum level. Bacteroidetes, Firmicutes and Proteobacteria were the dominant phyla in all of the group. However, there were differences in the composition of the flora at the genus level. The proportion of *Bacteroides* among *H. pylori* infected individuals decreased from 38.9% to 31.6% when compared with non-infected individuals, while the proportion of *Prevotella* increased from 5.0% to 10.8%.

*Bacteroides* species, as gut ‘friendly’ commensals, supply nutrients to other microbial residents of the gut [23]. Metabolites secreted by *Bacteroides* assist in maintaining stability of the immune system. These are important for the maintenance of intestinal homeostasis. *Bacteroides* decreasing after *H. pylori* infection may promote the intestinal microflora disorder. However, the recovery of the abundance of *Bacteroides* 42 days after eradication therapy also implied that eradication therapy, containing minocycline, metronidazole, bismuth agents and proton pump inhibitors, can effectively regulate intestinal homeostasis.

*Prevotella* is a genus of gram-negative anaerobic bacteria. According to the study of Jeppe et al., the increased *Prevotella* can activate Toll-like receptor 2, leading to the production of Th17-polarizing cytokines such as IL-23 and IL-1 by antigen-presenting cells. Furthermore, *Prevotella* stimulate epithelial cells to produce IL-8, IL-6 and CCL20, which can promote Th17 immune responses and neutrophil recruitment. *Prevotella*-mediated mucosal inflammation leads to systemic dissemination of inflammatory mediators, bacteria and bacterial products, which in turn may affect systemic disease outcomes[24]. The increase of *Prevotella* mediates the occurrence and development of *H. pylori*-related diseases to a certain extent. Same as *Bacteroides*, the abundance of *Prevotella* returned to healthy control level after eradication treatment containing minocycline, metronidazole, bismuth agents and proton pump inhibitors.

By Kruskal–Wallis rank sum test, it was found that *Entercoccus* was significantly increased in *H. pylori* infected patients, and increased again after 14 days of eradication treatment, but returned to normal level after 42 days of treatment. *Enterococcus* is commensal bacteria inhabiting the intestines of humans, which is the major conditionally pathogenic bacteria that cause hospital-acquired infections [25]. The current drug susceptibility test shows that the resistance rate of Enterococcus to minocycline was 87.1%, which may explain the significant increase of *Enterococcus* abundance in *H. pylori* infected individuals treated with minocycline regimen [26]. However, the result showed that the abundance of *Enterococcus* returned to normal level one month after drug withdrawal. Therefore, although *Enterococcus* is opportunistic pathogen, it is unnecessary to worry about *Enterococcus*-associated bacterial infections caused by the use of minocycline regimens.

In our research, *Muribaculaceae* only survived in *H. pylori* infected individuals before treatment and 42 days after treatment. And the abundance was significantly higher in *H. pylori* infected individuals before treatment than 42 days after treatment. *Muribaculaceae* was a newly discovered genus that was not reported until 2019 [27]. The current study suggested that *Muribaculaceae* contributed to propionate production, which was a kind of short-chain fatty acid (SCFAs) [28]. SCFAs can reduce the pH, promote the growth and proliferation of probiotics, and inhibit the reproduction of specific pathogenic bacteria. SCFAs have a positive effect on metabolic health [29]. Studies show SCFAs as substrates for energy production, lipogenesis, gluconeogenesis and cholesterol synthesis [30, 31]. In Wang's study, intestinal dysbiosis could be reversed by increasing the content of *Muribaculaceae* in the intestinal flora [32]. Therefore, the growth of *Muribaculaceae* may be able to resist the damage caused by *H. pylori*, and can regulate the intestinal ecological environment. Furthermore, carbohydrate metabolism genes were up-regulated in *Muribaculaceae*, which may explain the elevated metabolism of starch/sucrose in *H. pylori* infected individuals.

Whether *H. pylori* can migrate from the stomach to the intestine is still suspended. In our research, *H. pylori* was not detected in any of the 60 stool samples in the *H. pylori*-infected individuals, which is consistent with several previous research. Iino et al. found that even in the subjects with *H. pylori* infection, the presence of *H. pylori* in the intestine is rare [33]. Therefore, we speculate that *H. pylori* may be limited in the upper digestive tract, but not transfer into the lower digestive tract. And *H. pylori* influence the intestinal flora through indirect effect. According to previous studies, the indirect effects of *H. pylori* on the intestine are as follows. Firstly, cytotoxin-associated gene A (Cag A), a virulence factor secreted by *H. pylori*, can induce intestinal microbial ecological imbalance [34]. Besides, *H. pylori* may trigger host immune response to influence the intestinal flora [35]. Thirdly, chronic *H. pylori* infection may change the acidic environment in the stomach, inducing more microorganisms reach the distal intestine by breaking through the gastric acid barrier [36]. But the exact mechanism of *H. pylori* affecting the intestinal flora is still suspended, perhaps further research on the metabolites of intestinal bacteria may answer this question.

In patients treated with eradication regimens of amoxicillin, clarithromycin, metronidazole, and lansoprazole, the diversity and composition of intestinal microbiota gradually return to baseline after 1 year [20]. In another research, the intestinal microbiota did not return to baseline until three months after triple therapy with vonoprazan, amoxicillin, and clarithromycin [21]. However, the minocycline regimen used in our research can restore the intestinal microbiota to baseline one month after drug withdrawal. Compared with previous studies, quadruple anti-*H. pylori* regimen containing minocycline and metronidazole has less impact on the intestinal flora and can restore the normal structure of the flora in a short period of time.

This study had several limitations. First, the sample size was relatively small. Perhaps increasing the sample size may show more statistically significant results. Second, the follow-up period of our research was short. The further research is needed on the long-term effects of minocycline regimen on intestinal microbiota. Third, we only analyzed the intestinal flora of patients who were successfully eradicated. Perhaps more meaningful results may be obtained by analyzing the intestinal flora of patients who failed to eradicate, and comparing with the patients successfully eradicated. Fourth, we only included patients from 25 to 50 years old. The benefit of eradicating *H. pylori* is the largest before precancerous lesions happening, such as intestinal metaplasia. So, the eradication of *H. pylori* in patients aged 25–50 years is of the greatest significance. However, patients of all ages should be included in scientific research.

In conclusion, intestinal microbiota diversity, composition, functional predictions altered significantly after *H. pylori* infection, and gradually returned to healthy control levels after the application of eradication therapy containing minocycline and metronidazole in one month and a half.

## Supplementary Information

**Additional file 1: Supplementary Figure 1.** **a** The resulting rarefaction curves indicate that microbial richness was near saturation at the applied sequencing depth. **b** The Shannon curves indicate that sequencing depth was adequate.

## Data Availability

The 16S rRNA sequence data generated in this study have been deposited in the Sequence Read Archive database under accession number PRJNA824269. The URL is as follows: https://trace.ncbi.nlm.nih.gov/Traces/sra/. Now the data has been uploaded but not been public. The data will not be available until paper being accepted.
