# Supplementary material for: The impact of Helicobacter pylori infection and eradication therapy containing minocycline and metronidazole on intestinal microbiota
Source: BMC Microbiol. 2022 Dec 29;22:321. doi: 10.1186/s12866-022-02732-6 (PMC9798553; doi:10.1186/s12866-022-02732-6)
Supplement: Supplementary file 1 — Additional file 1: Supplementary Figure 1. a The resulting rarefaction curves indicate that microbial richness was near saturation at the applied sequencing depth. b The Shannon curves indicate that sequencing depth was adequate. [file 12866_2022_2732_MOESM1_ESM.docx]

Supplementary Figure 1. (a) The resulting rarefaction curves indicate that microbial richness was near saturation at the applied sequencing depth. (b) The Shannon curves indicate that sequencing depth was adequate.
